# Intrarenal neurofibroma: unveiling a diagnostic challenge—a case report and literature review

**DOI:** 10.1093/jscr/rjae285

**Published:** 2024-05-02

**Authors:** Mahmoud Mustafa, Abdelkarim Barqawi, Amir Aghbar, Ibraheem Alami, Honood Abu Ras

**Affiliations:** Department of Medicine, College of Medicine and Health Sciences, An-Najah National University, Al-Junied Street, Nablus 44839, Palestine; Department of Urology, An-Najah National University Hospital, Asira Street, Nablus 44839, Palestine; Department of Medicine, College of Medicine and Health Sciences, An-Najah National University, Al-Junied Street, Nablus 44839, Palestine; Department of General surgery, An-Najah National University Hospital, Asira Street, Nablus 44839, Palestine; Department of Medicine, College of Medicine and Health Sciences, An-Najah National University, Al-Junied Street, Nablus 44839, Palestine; Department of Urology, An-Najah National University Hospital, Asira Street, Nablus 44839, Palestine; Department of Medicine, College of Medicine and Health Sciences, An-Najah National University, Al-Junied Street, Nablus 44839, Palestine; Department of Urology, An-Najah National University Hospital, Asira Street, Nablus 44839, Palestine; Department of Medicine, College of Medicine and Health Sciences, An-Najah National University, Al-Junied Street, Nablus 44839, Palestine; Department of Pathology, An-Najah National University Hospital, Asira Street, Nablus 44839, Palestine

**Keywords:** neurofibroma, kidney tumor, benign, nephrectomy, case report

## Abstract

A 53-year-old male patient presented with an incidental finding of a left kidney mass after being evaluated for elevated serum creatinine without having any symptoms. The left kidney mass was confirmed by ultrasound, computed tomography ‘CT’ scan and magnetic resonance imaging ‘MRI’. A left radical nephrectomy was done, and histopathology confirmed the presence of intrarenal neurofibroma with no evidence of malignancy.

## Introduction

Neurofibroma is an uncommon benign tumor arising in peripheral nerves, and rarely occurs in the kidneys and parapelvic areas [1, 2]. Only eight cases were reported, the first of which was in 1967 [1–5]. A diagnostic dilemma has been faced in the preoperative diagnosis of kidney neurofibroma as it mimics renal cell carcinoma (RCC) or transitional cell carcinoma (TCC). Solitary neurofibroma occurs without a genetic, mutational or syndromic manifestations [1, 4]. In this report, we are presenting a case of a 53-year-old male patient with an incidental finding of a left kidney mass, who underwent radical nephrectomy, and the histopathological result was consistent with neurofibroma.

## Case report

A 53-year-old male patient with a past medical history of hypertension for 10 years, diabetes mellitus (DM) for 20 years and ischemic heart disease with previous percutaneous coronary intervention was incidentally found to have elevated serum creatinine (1.6 mg/dl) as part of routine follow-up evaluation for DM. The patient reported previous normal serum creatinine with no history of hematuria, abdominal pain, weight loss, anorexia or decreased oral intake. Moreover, there was no history of prior surgical or endourological interventions and no family history of kidney tumors.

**Figure 1:**
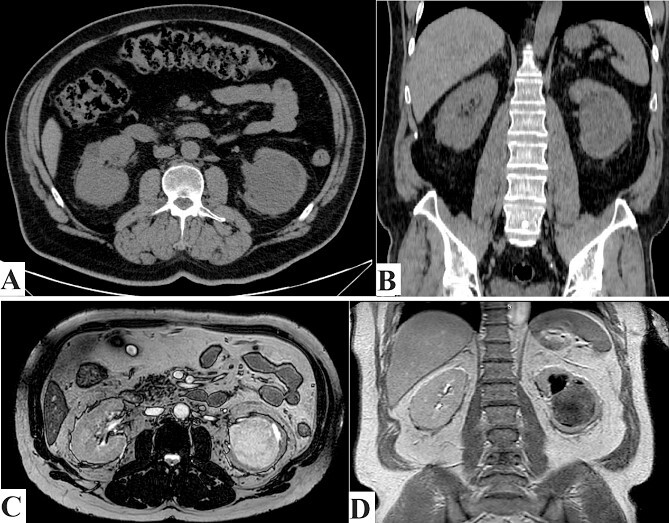
(A) CT scan axial section and (B) CT scan coronal section. Showing left kidney well-defined rounded soft tissue density mass lesions measuring about 6.7 cm in the lower aspect of the renal medulla. (C) MRI axial section, and (D) MRI coronal section. Showing left kidney large well defined rounded hypointense on T1W and heterogenous hyperintense on T2W images focus seen in the lower aspect of the medulla of the left kidney measuring about 6.8 cm approximately, resulting in mild calyceal dilatation.

As part of his evaluation for his elevated serum creatinine, abdominal ultrasound showed the presence of a large, well-defined hypoechoic medullary lesion with internal heterogeneity and minimal vascularity measuring about 6.7 × 7.4 cm^2^ and abutting the inner aspect of the left renal cortex. Renal computed tomography (CT) scan without contrast demonstrated a well-defined rounded soft tissue density mass lesion measuring about 6.7 × 6.4 × 7.9 cm^3^ in the lower aspect of the left renal medulla (Fig. 1A and B).

As the serum creatinine was elevated, preventing the use of contrast material with the CT scan, abdomen magnetic resonance imaging ‘MRI’ with gadolinium IV contrast showed a large, well-defined rounded lesion, hypointense on T1W and heterogeneous hyperintense on T2W image, with a focus seen in the lower aspect of the medulla of the left kidney measuring about 6.8 × 7.5 cm^2^ approximately, resulting in mild calyceal dilatation. After IV contrast administration, it showed mild heterogeneous enhancement, more marked in the delayed images. Two para-aortic small solid lymph nodes, the biggest measuring 1.2 cm, were detected. Chest imaging showed no evidence of distant metastasis (Fig. 1C and D).

**Figure 2:**
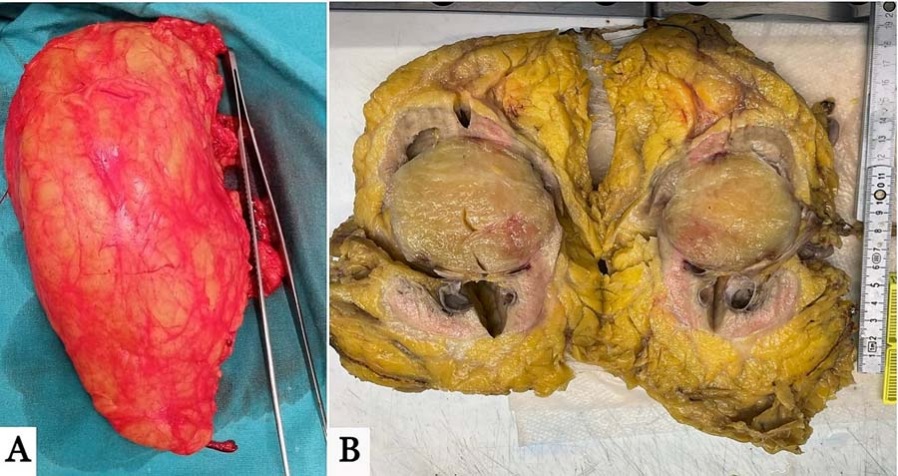
(A) Gross images of the harvested left kidney with the tumor located in the lower pole. (B) Cross-section of the harvested specimen showing a well-demarcated circumscribed mass located inside renal parenchyma at the lower aspect of the left renal medulla.

**Figure 3:**
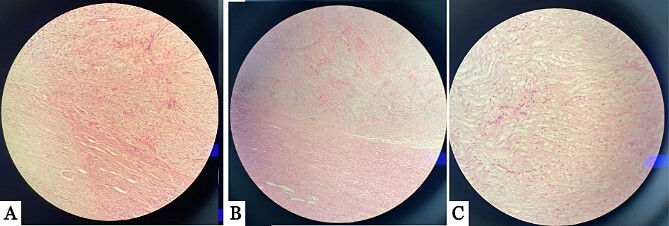
Multiple histopathology sections showing intrarenal neurofibroma with normal kidney tissues. (A) and (B) show the tumor located inside renal parenchyma, juxta to the renal cortical parenchyma. (C) The image show spindle cell tumor with serpentine wavy nuclei arranged in a fascicular pattern.

The left kidney mass was suspicious for malignancy, for which the patient underwent left radical nephrectomy with the removal of the suspicious para-aortic lymph nodes (Fig. 2). The histopathology report showed a final diagnosis of neurofibroma with no malignancy. Para-aortic lymph nodes were also free of malignancy. Histopathological description of the sample showed a 6 × 6 × 5 cm^3^ tumor that was located in the lower pole of the removed kidney, encapsulated with yellowish cut surfaces, and was limited to the kidney and did not invade the Gertoa’s fascia. Tumor cells were focally positive for S100 and negative for smooth muscle actin ‘SMA’ and desmin. The pathological findings in the remaining part of the kidney included glomerulosclerosis, interstitial fibrosis and tubular atrophy (Fig. 3). Neurofibroma in our case was truly intrarenal, and this can be demonstrated in Figs 2 and 3, where tumorous tissues appear adjacent to normal kidney tissues.

## Discussion

Neurofibroma is a benign tumor rarely affecting the kidney, and few cases have been reported worldwide [1]. They tend to be solitary, localized and circumscribed [2]. Different clinical presentations have been described for the few reported cases of neurofibroma, ranging from no symptoms to flank pain or hematuria (depending on the lesion's extent within the kidney). All of the reported cases of renal or parapelvic neurofibroma had a preoperative diagnostic challenge in confirming the nature of the kidney mass, given its imaging resemblance to RCC or TCC, and thus all of the cases were treated radically [1]. Involvement of renal sinuses, calyces or upper ureters has also been reported, making preoperative diagnosis more challenging.

In our case, there was a suspicion of RCC based on preoperative imaging, including a CT scan and MRI, which favored our decision to proceed with radical nephrectomy. To our knowledge, most of the reported cases in the literature have also been managed similarly, given the diagnostic dilemma of preoperative diagnosis. Therefore, histopathological examination remains the only way to establish the diagnosis of kidney neurofibroma [4].

After reviewing all of the eight reported cases [1–5], there was no radiological suspicion related or specific to neurofibroma during the standard preoperative imaging techniques. Except for two patients who presented with hematuria, the remaining patients presented with pain. In our case, the patient’s diagnosis was incidental. The average age of presentation in all the reported cases ranged from 33 to 59 years, with no predominance in certain genders (four males, three females, and one unknown gender). The volume of the lesions in the reported cases did not exceed 10 cm, which did not raise any special suspicion during the workup, for which there was no deviation from the standard preoperative evaluation for renal RCC. Regarding the location of the tumor, in six out of the eight reported cases, the tumor was located in the renal sinus; the remaining two were located in the retroperitoneum and lower pole. In our case, the lesion was located in the lower aspect of the left renal medulla. Thus, a high index of suspicion is required for any patient with a mass in the renal sinus; ureteroscopy with or without biopsy should be done to distinguish the upper TCC from other renal masses, thus avoiding unnecessary ureterectomy and bladder cuff excision if TCC is ruled out.

Histologically, the examined tumor in the removed kidney in our patient showed an encapsulated lesion with yellowish cut surfaces and was limited to the kidney and did not invade the Gertoa’s fascia. Tumor cells were focally positive for S100 and negative for SMA and desmin, making other mesenchymal tumors, such as solitary fibrous tumor, unlikely. Thus, immunohistochemistry is vital in formulating a final diagnosis [2].

## Conclusion

Neurofibroma of the kidneys is a rare benign tumor with a limited propensity to affect the kidney and parapelvic spaces. No imaging pathognomic findings have been described to help differentiate it from RCC or TCC, making diagnosis challenging and based mainly on histopathological examination. However, a preoperative ureteroscopy with biopsy may determine the nature of the tumor, thus avoiding unnecessary nephrectomy.

## Author contributions

All authors made substantial contributions to conception and design. They have all agreed to submit to the current journal; gave final approval of the version to be published; and agreed to be accountable for all aspects of the work.

## Conflict of interest statement

None declared.

## Funding

This research did not receive any kind of funds.

## Consent

A written signed consent was obtained from the patient for the purpose of this article publication and its attached images.
